# The structure of a complex of the lactonohydrolase zearalenone hydrolase with the hydrolysis product of zearalenone at 1.60 Å resolution

**DOI:** 10.1107/S2053230X17007713

**Published:** 2017-06-17

**Authors:** Qi Qi, Wen-Jing Yang, Hu-Jian Zhou, Deng-Ming Ming, Kai-Lei Sun, Tian-Yu Xu, Xiao-Jian Hu, Hong Lv

**Affiliations:** a State Key Laboratory of Genetic Engineering, School of Life Sciences, Fudan University, Shanghai 200438, People’s Republic of China; b Shanghai Engineering Research Center of Industrial Microorganisms, Shanghai 200438, People’s Republic of China; c Shanghai Collaborative Innovation Center for Biomanufacturing Technology, Shanghai 200237, People’s Republic of China

**Keywords:** lactonohydrolase, *Clonostachys rosea*, zearalenone, catalysis mechansim

## Abstract

Zearalenone hydrolase (ZHD) is an α/β-hydrolase that detoxifies and degrades the lactone zearalenone (ZEN), a naturally occurring oestrogenic mycotoxin that contaminates crops. Several apoenzyme and enzyme–substrate complex structures have been reported in the resolution range 2.4–2.6 Å. However, the properties and mechanism of this enzyme are not yet fully understood. Here, a 1.60 Å resolution structure of a ZHD–product complex is reported which was determined from a C-terminally His_6_-tagged ZHD crystal soaked with 2 m*M* ZEN for 30 min. It shows that after the lactone-bond cleavage, the phenol-ring region moves closer to residues Leu132, Tyr187 and Pro188, while the lactone-ring region barely moves. Comparisons of the ZHD–substrate and ZHD–product structures show that the hydrophilic interactions change, especially Trp183 N^∊1^, which shifts from contacting O2 to O12′, suggesting that Trp183 is responsible for the unidirectional translational movement of the phenol ring. This structure provides information on the final stage of the catalytic mechanism of zearalenone hydrolysis.

## Abstract 2

The high-resolution crystal structure of a complex of the lactonohydrolase zearalenone hydrolase with the hydrolytic product of the oestrogenic myotoxin zearalenone provides information on the final stage of the catalytic mechanism.

## 1. Introduction   

Recent studies regarding the hydrolysis of the lactone zearalenone (ZEN) have attracted great interest in the field and have promoted efforts to identify lactonases with high efficiency, since such enzymes may provide invaluable applications in the field of food safety (Dong *et al.*, 2001 ▸; Takahashi-Ando *et al.*, 2002 ▸; Bains *et al.*, 2011 ▸). ZEN is a resorcylic acid lactone that is produced by several *Fusarium* species as an oestrogenic mycotoxin (Pittet, 1998 ▸). ZEN is known to contaminate crops, in addition, upon ingestion ZEN has been shown to cause oestrogenic syndrome in livestock and humans by damaging the reproductive system (Lindsay, 1985 ▸; Mirocha *et al.*, 1971 ▸). ZEN is a heat-stable molecule that survives the cooking process (Winssinger & Barluenga, 2007 ▸), and is difficult to remove from cereal crops (Kuiper-Goodman *et al.*, 1987 ▸; Tanaka *et al.*, 1988 ▸).

Zearalenone hydrolase (ZHD) is an α/β-hydrolase that has been shown to be able to detoxify ZEN (Takahashi-Ando *et al.*, 2002 ▸, 2004 ▸). ZHD has demonstrated potential for use as a treatment for ZEN contamination that will not result in damage to cereal crops. Structures of apo ZHD and some enzyme–substrate complexes have recently been reported (Peng *et al.*, 2014 ▸; Xu *et al.*, 2016 ▸). These structures reveal a catalytic triad consisting of Ser102–His242–Glu126 inside a ZEN-binding tunnel that is enclosed by the α/β-hydrolase fold and a helical cap domain. This catalytic triad in ZHD is consistent with the generic catalytic motif of the α/β-hydrolase family. It is typically comprised of a nucleophilic residue (Ser, Cys or Asp), the general acid–base catalyst His and an acidic residue (Glu or Asp) (Ollis *et al.*, 1992 ▸; Lenfant *et al.*, 2013 ▸; Heikinheimo *et al.*, 1999 ▸). However, the properties and mechanism of this enzyme are not yet fully understood. Here, we report the 1.60 Å resolution structure of a complex of ZHD with the hydrolytic product of zearalenone, which reveals the final stage of zearalenone hydrolysis.

## 2. Materials and methods   

### 2.1. Macromolecule production   

The ZHD sequence was cloned from the genomic DNA of a *Clonostachys rosea* strain (purchased from China General Microbiological Culture Collection Center; CGMCC). The clone encodes eight amino-acid variations compared with the sequence in GenBank (ALI16790.1). All eight varying amino acids, Val26Ile, Pro69Ala, Val87Ile, Lys148Asn, Met168Leu, Asp170Val, Lys198Gln and Leu200Val, are located on the surface of the enzyme and are distant from the catalytic pocket. The ZHD gene was inserted into the pET-28b(+) vector using the NcoI and EcoRI restriction enzymes. The recombinant ZHD protein was connected to a His_6_ tag through a 14-amino-acid linker at the C-terminus (Table 1 ▸). After induction with 0.2 m*M* IPTG, recombinant protein was overexpressed in *Escherichia coli* BL21 (DE3) cells at 18°C for 20 h. The protein was then purified using an Ni–IDA column followed by a HiTrap Q HP column, and finally by a HiLoad 16/60 Superdex 200 prep-grade column (GE Healthcare, USA). The elution position from the Superdex column suggested that ZHD is a dimer in solution.

The purified ZHD protein was extensively dialysed and concentrated in a buffer consisting of 20 m*M* imidazole pH 7.8, 300 m*M* KCl, 2 m*M* dithiothreitol (DTT), 10 m*M* ammonium dibasic phosphate, 5%(*v*/*v*) glycerol. The measurement of the catalytic activity was based on literature methods (Takahashi-Ando *et al.*, 2004 ▸; Peng *et al.*, 2014 ▸) with minor modifications. The total 50 µl reaction volume contained 15 n*M* ZHD and 1–50 µ*M* ZEN (Sigma) in 100 m*M* Tris–HCl pH 9.5 buffer. After 0–32 min at 30°C, the reaction was terminated by adding 50 µl 200 m*M* HCl. Following centrifugation at 12 000*g*, 20 µl of sample was analyzed by high-performance liquid chromatography (HPLC; Agilent 1260 Infinity equipped with an Agilent C18 chromatography column). The amount of ZEN remaining was calculated from the integral of the peak area.

### 2.2. Crystallization and zearalenone-soaking experiment   

Crystallization conditions for ZHD are summarized in Table 2 ▸. Rod-shaped crystals appeared within 2–3 d and had dimensions of approximately 2 × 0.3 × 0.2 mm. Prior to cooling in liquid nitrogen, crystals were transferred to a cryoprotectant solution consisting of 600 m*M* ammonium dibasic phosphate, 100 m*M* KCl, 50 m*M* imidazole pH 7.4, 30%(*v*/*v*) glycerol for 10 s. In the enzyme–product complex soaking experiments, the harvested ZHD crystals were soaked in cryoprotectant with an additional 2 m*M* ZEN for 30 min.

### 2.3. Data collection and processing   

All diffraction data were collected at −173°C on the BL17U1 beamline at SSRF (Wang *et al.*, 2015 ▸). *HKL*-2000 was used for data indexing, integration and scaling (Otwinowski & Minor, 1997 ▸). The data-collection statistics are shown in Table 3 ▸.

### 2.4. Structure solution and refinement   

We first determined the apoenzyme structure using the single-wavelength anomalous diffraction (SAD) method with a selenomethionine derivative (PDB entry 5xmw). Although there are differences in the space group and molecular packing pattern, this showed that our structure is identical to that reported previously (Peng *et al.*, 2014 ▸). We used our apo structure as the initial molecular-replacement model for determination of the enzyme–product complex structure. Molecular replacement was performed with *Phaser* (McCoy *et al.*, 2007 ▸). Structure refinement, which is summarized in Table 4 ▸, was performed with *PHENIX* (Adams *et al.*, 2010 ▸), *REFMAC*5 (Murshudov *et al.*, 2011 ▸), *CCP*4 (Winn *et al.*, 2011 ▸) and *Coot* (Emsley *et al.*, 2010 ▸).

## 3. Results and discussion   

**Figure 1:**
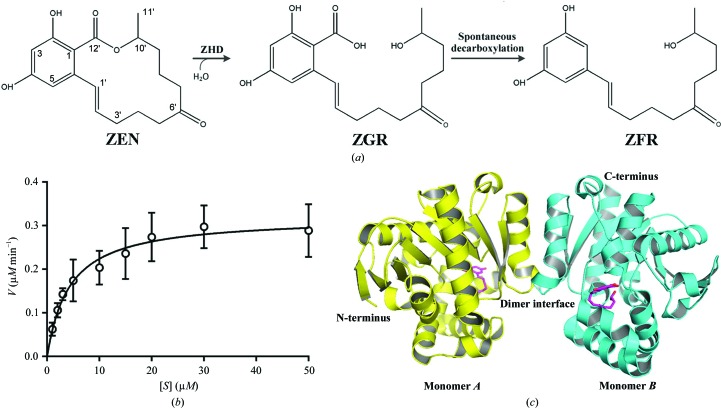
Hydrolysis of zearalenone (ZEN) by ZHD. (*a*) The ZEN hydrolysis reaction. ZEN is hydrolysed by ZHD to ZGR. ZGR then spontaneously decarboxylates to ZFR in solution. (*b*) The catalytic activity of ZHD. Assays were carried out in triplicate. (*c*) Two ZHD molecules packed into one asymmetric unit of the ZHD–ZGR complex.

ZEN is comprised of a phenol ring and a large lactone loop (Fig. 1 ▸ *a*). ZHD has been shown to cleave the intramolecular ester bond within ZEN, converting it to the less toxic 2,4-dihydroxy-6-[(1*E*,10*S*)-10-hydroxy-6-oxoundec-1-en-1-yl]­benzoic acid (ZGR; Fig. 1 ▸ *a*). The carboxyl group of this molecule spontaneously leaves in alkaline solution, resulting in the formation of (1*E*,10*S*)-1-(3,5-dihydroxyphenyl)-10-hydroxy­undec-1-en-6-one (ZFR) (El-Sharkawy & Abul-Hajj, 1988 ▸; Takahashi-Ando *et al.*, 2002 ▸). Compared with conventional nucleophilic hydrolases, the catalytic activity of ZHD for the hydrolysis of ZEN was very low. When purified C-terminally His_6_-tagged ZHD was incubated with ZEN at pH 9.5 (Fig. 1 ▸ *b*), the *k*_cat_ and *k*_cat_/*K*_m_ were determined to be 0.3 s^−1^ and 8.5 × 10^4^ s^−1^ *M* ^−1^, respectively. Compared with the reported parameters at the same pH, the *k*_cat_ values are equal and our *K*_m_ value is a little lower (3.5 *versus* 16.7 µ*M*; Takahashi-Ando *et al.*, 2004 ▸).

The crystals of our C-terminally His_6_-tagged ZHD protein diffracted to approximately 1.60 Å resolution, which is much better than the resolutions reported from structures crystallized using an N-terminally His_6_-tagged ZHD construct (Peng *et al.*, 2014 ▸). In our structure the helices from Pro217 to Ala231 were found to form a dimeric interface through hydrophobic interactions (Fig. 1 ▸ *c*), which is identical to the previously reported structures.

**Figure 2:**
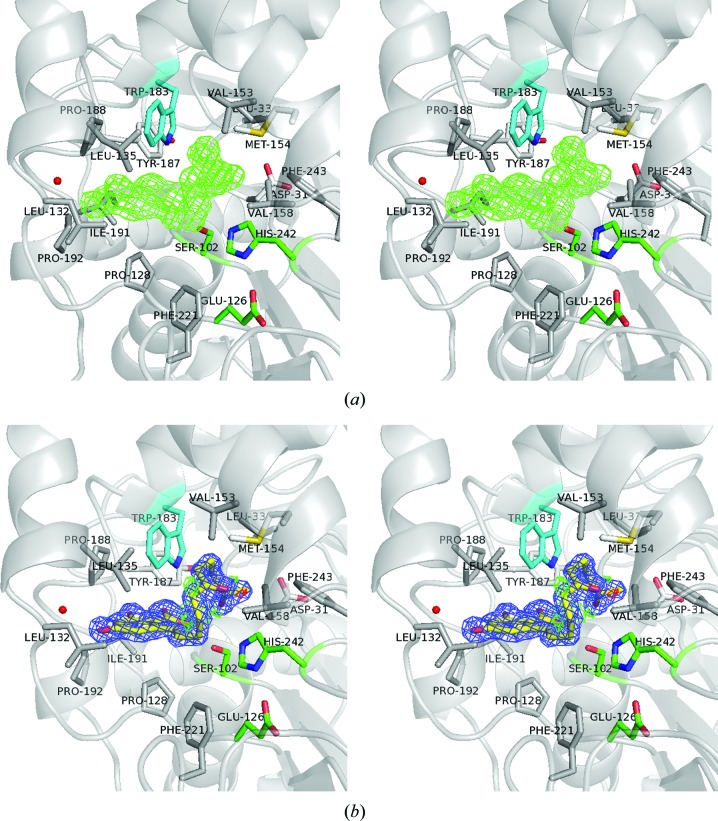
The ZGR molecule in the substrate-binding pocket. (*a*) Wall-eyed stereo presentation of the initial difference map. The *F*_o_ − *F*_c_ difference density OMIT map contoured at 3.0σ (green) clearly shows the skeleton of ZGR. (*b*) Wall-eyed stereo presentation of the ZGR structure. The 2*F*_o_ − *F*_c_ electron-density map contoured at 1.5σ is shown in blue. The *F*_o_ − *F*_c_ map contoured at ±3.0σ is shown in green and red, respectively. The catalytic triad Ser102–His242–Glu126 is shown in green. The environment surrounding ZGR shows the hydrophobic interactions with amino acids (grey sticks) and the hydrophilic interactions of ZGR with Ser102, Trp183 (in cyan) and one water molecule (red sphere).

In order to obtain the enzyme–product complex structure, apo ZHD crystals were soaked in 2 m*M* ZEN for between 1 and 45 min before quenching in liquid nitrogen. The expected hydrolytic product ZGR did not appear until the crystals had been soaked for over 30 min. In the structure of the ZHD–ZGR complex (PDB entry 5c8z), the product exhibited extensive hydrophobic contacts with surrounding amino acids in the binding pocket. These hydrophobic residues included Asp31, Leu33, Pro128, Leu132, Leu135, Val153, Met154, Val158, Trp183, Tyr187, Pro188, Ile191, Pro192, Phe221 and Phe243 (Fig. 2 ▸). The conjugated coplanar structure of ZGR was comprised of an entire phenol ring, an adjacent carbon double bond C1′=C2′ and a carboxyl group O12′=C12′—O13′. This carboxyl group in ZEN, which is present in the ZHD/S102A–ZEN complex structure (PDB entry 3wzm; Peng *et al.*, 2014 ▸), was not in a perfect coplanar orientation with the phenol-ring structure. This is likely to be owing to the internal tension imposed by the closed lactone loop, as well as the tight contacts within the catalytic pocket. When the product (ZGR) complex was superimposed on the substrate (ZEN) complex, changes in the phenol-ring position were noted. In the product complex the phenol ring of ZGR moves closer to residues Leu132, Tyr187 and Pro188 (Figs. 2 ▸ and 3 ▸ *a*).

**Figure 3:**
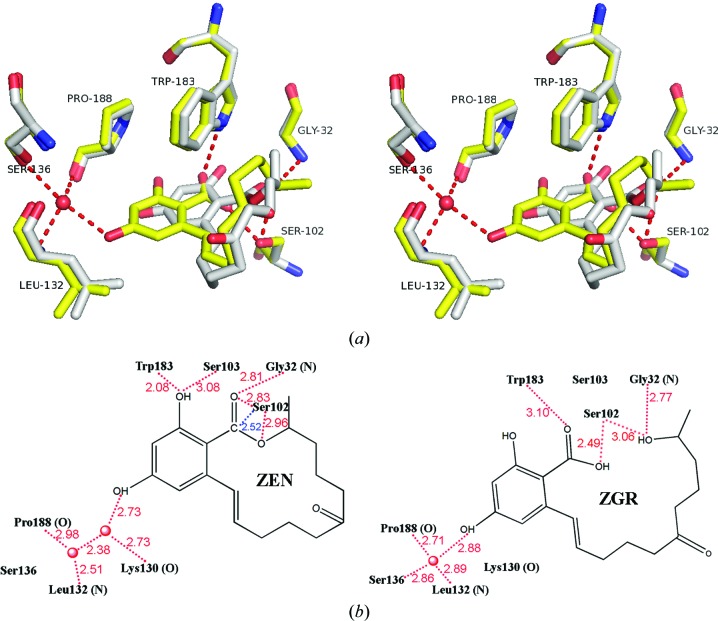
The hydrophilic interaction between ZHD and ZGR. (*a*) Wall-eyed stereo presentation of the hydrophilic interaction in the ZGR (yellow) binding pocket. The red dotted lines indicate hydrogen bonds. The ZEN (white) and surrounding hydrophilic interacting amino acids are superimposed without the two water molecules. (*b*) A comparison of the hydrophilic interactions in ZHD–ZEN (left, plotted according to PDB entry 3wzm and with Ala102 superimposed with Ser) and ZHD–ZGR (right). Red dashed lines indicate hydrogen bonds. Numbers indicate distances in Å. The blue line shows the potential nucleophilic attack by Ser102.

Comparing the hydrophilic interactions in the ZHD–ZGR and ZHD–ZEN complexes, we note differences involving interactions with water molecules (Fig. 3 ▸ *b*). Specifically, in the case of the ZHD/S102A–ZEN structure two water molecules were found in the pocket and formed hydrogen bonds to Lys130 O, Leu132 N, Pro188 O and ZEN O4. In the same area of our ZHD–ZGR complex, only one water molecule was found to remain, which formed hydrogen bonds to ZGR O4, Ser136 O^γ^, Leu132 N and Pro188 O (Fig. 3 ▸ *b*). When the hydrolysis of ZEN begins, cleavage of the ester bond helps to release the stress enclosed within the lactone loop. The phenol ring swings and pushes one of the two water molecules out of the pocket, until the remaining water molecule is stopped by Ser136 O^γ^.

Other critical hydrophilic interactions were observed near the lactone bond that could play a role in the catalytic mechanism. Structural superposition of the ZHD/S102A–ZEN structure and our ZHD–ZGR structure revealed that Ser102 O^γ^ of the catalytic triad points towards the lactone C atom C12′ of the carboxyl group at a distance of 2.52 Å. This suggests that a nucleophilic attack on C12′ initiates the hydrolysis reaction. This initial conformation was also found to be stabilized by Gly32 N–ZEN O12′, Trp183 N^∊1^–ZEN O2 and Ser103 O^γ^–ZEN O2 hydrogen bonds. Here, it seems that Ser103 O^γ^ is non-essential, as when it is mutated to Ala the catalytic activity is unaffected (Peng *et al.*, 2014 ▸). In the ZHD–ZGR complex, we found that Ser102 O^γ^ shifts to make contact with ZGR O13′, Gly32 N shifts to make contact with ZGR O10′, and Trp183 N^∊1^ shifts to make contact with ZGR O12′ (Fig. 3 ▸ *b*). Taken together, we suggest that these interactions are altered as a result of the breakage of the lactone bond. It is very likely that Trp183 N^∊1^ is responsible for the unidirectional translational movement of the phenol ring, which in turn determines the conformational change of the lactone loop following cleavage of the lactone bond. When Trp183 was mutated, the enzymatic activity was found to be dramatically decreased (Peng *et al.*, 2014 ▸).

Thus far, two distinct mechanisms have been proposed for the hydrolysis reaction of the α/β-hydrolase family. The first is a nucleophilic mechanism that typically begins with the serine attacking the carbonyl C atom of the substrate, which forms the iconic tetrahedral intermediate. This mechanism is strongly supported by observations of trapped acyl-enzyme intermediates (Ding *et al.*, 1994 ▸; Hedstrom, 2002 ▸; Ruzzini *et al.*, 2012 ▸). This nucleophilic mechanism was recently used to explain hydrolysis by the *N*-acyl homoserine lactonase AidH (Gao *et al.*, 2013 ▸). The second hydrolysis mechanism is typically referred to as the general base mechanism. This typically begins with substrate deprotonation (Sun *et al.*, 2014 ▸; Wajant & Pfizenmaier, 1996 ▸; Zuegg *et al.*, 1999 ▸). In the case of ZHD, this enzyme–product structure provides the final state of catalysis. However, it is difficult to infer the corresponding hydrolysis mechanism, as detailed structures of the enzyme intermediates are still lacking.

## Supplementary Material

PDB reference: ZHD–ZGR complex, 5c8z

PDB reference: apo ZHD, selenomethionine derivative, 5xmw

## Figures and Tables

**Table 1:** Macromolecule-production information for ZHD

Source organism	*C. rosea*
DNA source	Genomic DNA
Forward primer†	5′-ATACCATGGACATGCGCACTCGTAGCACAA-3′
Reverse primer‡	5′-CTCGAATTCCAAAGATGCTTCTGCGTAGTT-3′
Cloning vector	pET-28b(+)
Expression vector	pET-28b(+)
Expression host	*E. coli* BL21 (DE3)
Complete amino-acid sequence of the construct produced§	(MD)MRTRSTISTPNGITWYYEQEGTGPDIVLVPDGLGECQMFDSSVSQIAAQGFRVTTFDMPGMSRSAKAPAETYTEVTAQKLASYVISILDALDIKHATVWGCSSGASTVVALLLGYPDRIRNAMCHELPTKLLDHLSNTAVLEDEEISNILANVMLNDVSGGSEAWQALGVEVHARLHKNYPVWARGYPRTIPPSAPVQDVEALRGKPLDWTVGAATPTESFFDNIVTATKAGVNIGLLPGMHFPYVSHPDVFAKYVVETTQKHL(WNSSSVDKLAAALEHHHHHH)

† The NcoI site sequence is underlined.

‡ The EcoRI site sequence is underlined.

§ The artificial additional amino acids are in parentheses and the C-terminal His tag is underlined.

**Table 2:** Crystallization conditions for ZHD

Method	Hanging-drop vapour diffusion
Plate type	24-well plate
Temperature (K)	293
Protein concentration (mg ml^−1^)	30.0
Buffer composition of protein solution	20 m*M* imidazole pH 7.8, 300 m*M* KCl, 2 m*M* DTT, 10 m*M* ammonium dibasic phosphate, 5%(*v*/*v*) glycerol
Composition of reservoir solution	1.2 *M* ammonium dibasic phosphate, 200 m*M* KCl, 100 m*M* imidazole pH 7.4
Volume and ratio of drop	1 µl, 1:1
Volume of reservoir (ml)	1

**Table 3:** Data-collection and processing statistics Values in parentheses are for the outer shell.

Diffraction source	BL17U1, SSRF
Wavelength (Å)	0.97923
Temperature (K)	100
Detector	ADSC Quantum 315r
Rotation range per image (°)	1
Total rotation range (°)	180
Exposure time per image (s)	2
Space group	*P*2_1_2_1_2_1_
*a*, *b*, *c* (Å)	74.90, 89.59, 113.79
α, β, γ (°)	90, 90, 90
Mosaicity (°)	0.32
Resolution range (Å)	50–1.60 (1.69–1.60)
Total No. of reflections	719066 (104738)
No. of unique reflections	101351 (14615)
Completeness (%)	99.9 (100.0)
Multiplicity	7.1 (7.2)
〈*I*/σ(*I*)〉	15.1 (4.3)
*R*_r.i.m_	0.032 (0.170)
Overall *B* factor from Wilson plot (Å^2^)	22.28

**Table 4:** Structure solution and refinement Values in parentheses are for the outer shell.

Resolution range (Å)	48.03–1.60 (1.657–1.60)
Completeness (%)	99.80 (100)
σ Cutoff	2.0
No. of reflections, working set	101261 (3139)
No. of reflections, test set	5060 (175)
Final *R*_cryst_	0.153 (0.168)
Final *R*_free_	0.167 (0.185)
No. of non-H atoms	
Protein	4349
Ligand	83
Water	324
Total	4756
R.m.s. deviations	
Bonds (Å)	0.008
Angles (°)	1.15
Average *B* factors (Å^2^)	
Overall	19.1
Protein	17.9
Ligand	26.8
Water	32.5
Ramachandran plot	
Most favoured (%)	98.7
Allowed (%)	1.3
